# A Volumetric and Functional Connectivity MRI Study of Brain Arginine-Vasopressin Pathways in Autistic Children

**DOI:** 10.1007/s12264-017-0109-2

**Published:** 2017-03-03

**Authors:** Xiao-Jing Shou, Xin-Jie Xu, Xiang-Zhu Zeng, Ying Liu, Hui-Shu Yuan, Yan Xing, Mei-Xiang Jia, Qing-Yun Wei, Song-Ping Han, Rong Zhang, Ji-Sheng Han

**Affiliations:** 1 Neuroscience Research Institute, Peking University, Beijing, 100191 China; 2 Department of Neurobiology, School of Basic Medical Sciences, Peking University, Beijing, 100191 China; 3 Key Laboratory for Neuroscience, Ministry of Education/National Health and Family Planning Commission, Peking University, Beijing, 100191 China; 4 Radiology Department, Peking University Third Hospital, Beijing, 100191 China; 5 Department of Pediatrics, Peking University Third Hospital, Beijing, 100191 China; 6 Mental Health Institute, Peking University, Beijing, 100191 China; 7 Key Laboratory of Ministry of National Health and Family Planning Commission, Beijing, 100191 China; 8 Sunshine Friendship Rehabilitation Centre, Beijing, 100085 China

**Keywords:** Autism, Children, Arginine-vasopressin, MRI, Structure, Connectivity, Behavior

## Abstract

Dysfunction of brain-derived arginine-vasopressin (AVP) systems may be involved in the etiology of autism spectrum disorder (ASD). Certain regions such as the hypothalamus, amygdala, and hippocampus are known to contain either AVP neurons or terminals and may play an important role in regulating complex social behaviors. The present study was designed to investigate the concomitant changes in autistic behaviors, circulating AVP levels, and the structure and functional connectivity (FC) of specific brain regions in autistic children compared with typically developing children (TDC) aged from 3 to 5 years. The results showed: (1) children with ASD had a significantly increased volume in the left amygdala and left hippocampus, and a significantly decreased volume in the bilateral hypothalamus compared to TDC, and these were positively correlated with plasma AVP level. (2) Autistic children had a negative FC between the left amygdala and the bilateral supramarginal gyri compared to TDC. The degree of the negative FC between amygdala and supramarginal gyrus was associated with a higher score on the clinical autism behavior checklist. (3) The degree of negative FC between left amygdala and left supramarginal gyrus was associated with a lowering of the circulating AVP concentration in boys with ASD. (4) Autistic children showed a higher FC between left hippocampus and right subcortical area compared to TDC. (5) The circulating AVP was negatively correlated with the visual and listening response score of the childhood autism rating scale. These results strongly suggest that changes in structure and FC in brain regions containing AVP may be involved in the etiology of autism.

**Electronic supplementary material:**

The online version of this article (doi:10.1007/s12264-017-0109-2) contains supplementary material, which is available to authorized users.

## Introduction

Autism spectrum disorder (ASD) is a developmental disorder with a high incidence and the symptoms may be life-long. ASD is characterized by impaired social interaction and communication as well as repetitive behaviors. In the United States, ASD affects 14.7 per 1,000 (1/68) children [1], and an estimated prevalence of 4.1 per 1,000 has been reported in China [2]. Besides, the prevalence is three to five times higher in boys than girls worldwide [1, 3, 4].

Arginine-vasopressin (AVP) and oxytocin (OXT) are nonapeptides with similar primary sequences, mainly synthesized in the hypothalamus. In the last decade, AVP and OXT have attracted more attention as neuropeptides in the central nervous system [5, 6] in addition to their peripheral effects on vascular and uterine smooth muscles. In the brain, AVP and OXT are released through a paracrine process into extra-hypothalamic areas and along the axonal projections to remote nuclei including the amygdala and hippocampus [6, 7], both of which are believed to be involved in certain mental diseases. Several lines of evidence suggest that dysfunction of the AVP and/or OXT system in these areas may be involved in the pathogenesis of ASD. These peptides are known to be heavily involved in the regulation of complex social cognition and behaviors [8–11], and impairment in social interaction is one of the core symptoms in ASD. Both AVP and OXT have been shown to play roles in neural development, and their receptors are highly expressed and constantly remodeled in the immature brain [7, 12–14]. Besides, the distribution of their receptors and the function of AVP/OXT in regulating behaviors are sexually dimorphic, especially during brain development [15–18]. Similar sexual dimorphism has also been noted in children with ASD.

Pharmacological studies have provided strong evidence that interfering with the AVP/OXT system results in changes in social behaviors in normal individuals and affects the symptoms in patients with ASD. Results from brain-imaging studies in healthy people have shown that intranasal AVP enhances emotion processing [19], altruistic interaction [20], and social recognition [21]. Intranasal OXT has been reported to improve trust [22], emotion processing [23–26], altruistic interaction [20], and empathy [27] in healthy participants. Recent studies have demonstrated that intranasal OXT improves the ability to infer the social emotions of others in autistic adults [28] and enhances brain function in children with autism during judgments of social/non-social pictures [29].

However, few studies have explored the relationship between dysfunction of the AVP system and abnormalities of the structure or function of brain regions in autistic patients, especially in very young children. Our previous studies have demonstrated that transcutaneous electrical acupoint stimulation (TEAS) improves autistic behaviors in children, possibly by enhancing the action of the AVP system [30]. In addition, Carson and colleagues recently found that plasma AVP concentrations can be used to predict AVP levels in the cerebrospinal fluid of children, and might serve as a biomarker for social function in children with autism [31, 32]. Therefore, changes in plasma AVP may be used as a less invasive approach to monitoring changes in the central AVP level in children.

Based on these findings, we designed a study using volumetric and functional connectivity measures with magnetic resonance imaging (MRI) techniques in an effort to answer the following questions: (1) whether the nuclei (i.e., hypothalamus, amygdala, and hippocampus) containing AVP perikarya or terminals show morphological abnormalities in children with ASD compared with typically-developing children (TDC); (2) whether these structures have aberrant connections with other areas in children with ASD; and (3) whether brain dysfunction is correlated with changes in the plasma level of AVP. To our knowledge, this is the first study to quantitatively explore a possible association between circulating AVP and the findings from MRI in children with ASD.

## Materials and Methods

### Participants

This study was approved by Peking University Institutional Review Board (IRB00001052-13079). The purposes and procedures were described orally to the parents of all the children, with question-and-answer sessions. Each participant’s parent(s) gave informed consent. Children with ASD were recruited from the Sunshine Friendship Rehabilitation Center for Children with Autism, Beijing, China. Twenty-one children with ASD aged between three and five years were recruited. Nineteen of the 21 completed MRI scanning. Two were excluded before completion of MRI scanning: one with a history of epilepsy, and one who woke up during the scan. Of the 19 scanned children with ASD, 4 were excluded because of abnormal brain structure (one had cavum septum sellucidum, one had a cortical developmental issue, and two had myelin dysplasia). One child’s data were discarded in the pre-processing due to head motion (>3 mm or 3°). Thus, high-quality functional and structural MRI data were successfully acquired from a total of 14 children with ASD (12 boys and 2 girls). Fourteen age- and gender-matched TDC (10 boys and 4 girls) were recruited and completed scanning. All participants in the study were Han Chinese.

### Diagnoses and Assessments of ASD

All the participants with ASD met the conditions of the International Statistical Classification of Diseases and Related Health Problems, 10^th^ revision [33], and the Diagnostic and Statistical Manual of Mental Disorders IV-Text Revision [34]. The Childhood Autism Rating Scale (CARS)[35] was used in 13 of the autistic children (one missed the evaluation) by the same experienced psychiatrist (MXJ). Children with ASD were excluded if they also had other neurodevelopmental diseases or psychiatric disorders, or had a history of using antipsychotic drugs. TDC were screened by an experienced pediatric psychiatrist (JSZ) to exclude any mental disorders.

Both children with ASD and TDC were assessed for autistic symptoms by their parent(s) using the following scales, with the exception of one child with ASD whose questionnaire was technically invalid. (1) The Autism Behavior Checklist (ABC), includes a series of atypical behaviors in five subdomains: sensory, relating, body and object use, language, and social and self-help [35, 36]. (2) The Autism Spectrum Quotient Children’s Version (AQ-Child), consists of 50 descriptive statements designed to detect autistic traits. And these statements were designed to assess the phenotype in five areas: social skills, attention switching, attention to details, communication, and imagination [37, 38].

### Collection of Blood Samples

Blood samples from all autistic children who completed MRI scanning were collected by a pediatric nurse in the morning between 08:00 and 10:30, no more than 7 days after MRI scanning. To minimize the potential impact of eating and drinking, parents were asked to ensure that their child fasted overnight and no more than 100 mL of fluid was taken. Five milliliters of venous blood was drawn into an EDTA tube (Becton, Dickinson and Co., Franklin Lakes, NJ) containing 2500 KIU aprotinin (Sigma-Aldrich, St. Louis, MO) and gently mixed. The samples were kept on ice for 15–30 min and centrifuged at 1600 g for 15 min at 4 °C. The plasma samples were divided into Eppendorf tubes (700 μL/tube) and frozen at −80°C until assay.

### Imaging Data Acquisition

To keep the young child’s head still during scanning, children were sedated [39, 40] with oral chloral hydrate (50 mg/kg body weight; maximum dose, 1 g). The child was put into the scanner after falling asleep for ~10–15 min.

Images were acquired in a GE Discovery MR750 3.0T scanner at Peking University Third Hospital. T1-weighted anatomical images in the sagittal plane were collected with a 3D fast spoiled gradient echo sequence: repetition time (TR) = 4.9 ms, echo time (TE) = 2 ms, flip angle = 15°, field of view (FOV) = 240 mm, in-plane resolution = 1 × 1 mm^2^, slice thickness = 1 mm, 170 slices. Functional images were collected axially using a gradient-echo echo-planar sequence sensitive to BOLD contrast. The acquisition parameters were as follows: TR = 3,000 ms, TE = 20 ms, flip angle = 90°, FOV = 240 mm, in-plane resolution = 2 × 2 mm^2^, slice thickness = 3 mm, slice gap = 0.3 mm, 41 slices. Children with abnormal brain structures were excluded by two neuro-radiologists (YL and XZZ).

### Structural Imaging Analyses

Structural images were processed with SPM8 (http://www.fil.ion.ucl.ac.uk/spm) and VBM8 toolbox (http://dbm.neuro.unijena.de/vbm) within the MatLab R2014a programming environment (The MathWorks, Natick, MA). Since the participants were pre-school children, a customized template and tissue-probability map (TPM) were created for the subsequent voxel-based morphometric analysis. T1 data from 12 additional age-matched children (2–6 years) acquired with the same sequence were retrieved from the hospital database, giving a total of 40 children, in order to calculate the template and TPM. The customized TPM was calculated in MNI (Montreal Neurological Institute) space with the TOM8 toolbox (http://irc.cchmc.org/software/tom.php) [41]. In addition, an average DARTEL (Diffeomorphic Anatomical Registration Through Exponentiated Lie Algebra) template of all the 40 children in MNI space was constructed using the DARTEL suite in SPM8. The customized template was then implemented into VBM8 as the high-dimensional DARTEL approach, while the affine-registered gray matter (GM) segments were warped to the averaged template and modulated. Raw volumes for GM, white matter (WM), cerebrospinal fluid (CSF), and total intracranial volume (TIV) were calculated in native space.

The normalized GM images were smoothed for a kernel with 8-mm full-width-at-half-maximal (FWHM). The two-sample *t*-test was applied to compare the voxel-based gray matter density between groups. The single-voxel threshold was set at *P* <0.01 and a minimal cluster size of 626 voxels was used to correct for multiple comparisons. The corrected threshold was determined by 5000 Monte Carlo simulations (FWHM = 8 mm, cluster connection radius: rmm = 5, with a mask of whole-brain GM at a resolution of 1.5 × 1.5 × 1.5 mm^3^). Parts of the positive results were further analyzed for volume measurement.

Regions of interest (ROIs) including the amygdala, hippocampus, and hypothalamus were extracted from the T1-weighted original space of each participant, manually traced by an experienced rater (XJS) using 3D Slicer (http://www.slicer.org) [42]. The locations of ROIs are described in the supplementary material. There was good test-retest reliability for the amygdala (Cronbach’s α = 0.992), hippocampus (Cronbach’s α = 0.995), and hypothalamus (Cronbach’s α = 0.991).

### Functional Imaging Analyses

Pre-processing of the functional images was analyzed with SPM8. The first 10 volumes were discarded and the remaining volumes were then realigned and corrected for slice timing. Each child’s T1 and fMRI images were first normalized to the custom template, and the customized template was normalized to the default EPI (echo-planar imaging) template with a voxel size of 3 × 3 × 3 mm^3^. After that, the parameter was applied to the normalized T1 and fMRI images, and finally smoothed at FWHM = 8 mm. The inclusion criteria for head motion were <3 mm translation and 3° rotation. One child with ASD was excluded due to head motion.

The functional connectivity (FC) analyses were processed using the REST V1.8 toolkit (http://resting-fmri.sourceforge.net) [43]. To remove the linear trend and reduce the effect of low-frequency drift and high-frequency noise, de-trending and band-pass filtering (0.01–0.08 Hz) were applied. To further control the non-neural noise, the six head motion parameters, the WM signal, the CSF signal and the global mean signal [44] were regressed out as covariates.

Due to the prior significant VBM (voxel-based morphometry) results and knowledge of the structure-function relationships of the central AVP/OXT systems, the left amygdala and left hippocampus were set as seed points based on automated anatomical labeling [45] of template-defined regions. Voxel-wise functional connectivity was calculated for each seed point. Analyses of covariance were then performed in the two groups (TDC and ASD) with covariates of age and gender. Pearson’s correlation coefficients between the significant brain regions were computed and normalized to z-values using Fisher’s z transformation for further analyses.

The statistical maps were corrected for multiple comparisons using cluster thresholds performed in the AlphaSim module of the REST toolbox. As determined by 5,000 iterations of Monte Carlo simulation (single *P* value = 0.05, FWHM = 8 mm, cluster connection radius: rmm = 5, with a mask of whole-brain GM with a resolution of 3 × 3 × 3 mm^3^), the minimum cluster size was 259 voxels.

### Biochemical Analyses

Plasma AVP concentrations were determined by enzyme immunoassay (EIA) (Enzo Life Sciences, Plymouth Meeting, PA). After extraction of AVP with acetone and petroleum ether, the assay was performed according to the manufacturer’s instructions. The EIA was highly sensitive with a detection limit of 3.39 pg/mL for AVP. The cross-reactivity with related peptides in human was <0.001%. The *r*-values of standard curves for the assays were >0.999.

### Statistics

Analysis of the non-imaging statistical data was performed in IBM SPSS Statistics 20.0 and Amos 20.0 (IBM, Armonk, NY). The demographics (except for gender) and scale characteristics were all normally distributed (Tables S1 and S2) and were compared between the two groups using the independent-sample *t* test. Data are expressed as mean ± SD. Gender comparison was made using the χ^2^ test. Differences in global tissues (GM, WM, CSF, and TIV) and brain regions were assessed with multivariate analysis of variance. To explore associations between the functional connectivity and behavioral scores or biochemical indices, Pearson correlations were used for normally-distributed data and Spearman correlations were used for non-normally-distributed data.

A structural equation model was used to clarify the relationships among plasma neuropeptides, functional connectivities, and behaviors. A *P* value >0.05 indicates an acceptance of the assumptive model. The fitness of the model was assessed by the goodness-of-fit index (GFI) and adjusted GFI (AGFI) in which >0.9 was considered to indicate a good fit. A root-mean-square error of approximation (RMSEA) value of ~0.08 or less indicated a reasonable error of approximation.

## Results

### Demographic Characteristics and Behavioral Measurements

Children with ASD and TDC were matched for age (*t* = 1.465, *P* = 0.1548) and gender (χ^2^ = 0.849, *P* = 0.3570). Children with ASD had significantly higher scores in the ABC (*F* = 6.128, *P* <0.0001) and AQ-Child (*F* = 5.617, *P* <0.0001) than TDC (Table 1), as expected.

**Table 1:** Demographic characteristics and behavioral measurements of participants.

Variables	TDC	ASD	*t* or χ^2^	*P* value
Number of participants	14	14	NA	NA
Age range	3.0–5.5	2.9–5.0	NA	NA
Mean age ± SD	4.5 ± 0.74	4.1 ± 0.72	1.465^a^	0.1548
Gender (boy:girl)	10:4	12:2	0.849^b^	0.3570
CARS score (mean ± SD)	NA	35.38 ± 4.00	NA	NA
ABC score (mean ± SD)	10.71 ± 10.63	53.46 ± 22.97	6.128^a^	<0.0001
AQ-Child score (mean ± SD)	17.29 ± 4.92	29.84 ± 6.63	5.617^a^	<0.0001

*CARS* childhood autism rating scale, *ABC* autism behavior checklist, *AQ-child* autism spectrum quotient children’s version, *TDC* typically developing children, *ASD* autism spectrum disorder

^a^Independent-sample *t* test, *t* score

^b^χ^2^ test, χ^2^

### Correlations Between Plasma AVP and Symptoms

**Fig. 1:**
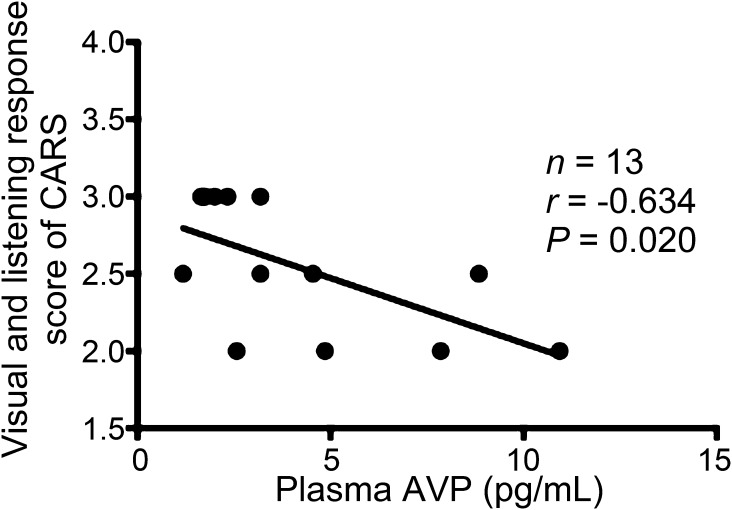
Correlations between CARS score and plasma AVP level in children with ASD. The visual and listening response score of CARS was negatively correlated with plasma AVP concentration. AVP, arginine-vasopressin; CARS, Childhood Autism Rating Scale.

Spearman correlations were used to explore the correlations between the plasma concentrations of the neuropeptide and behavioral scores. The plasma AVP level was negatively correlated with the visual and listening response score (*r* = −0.634, *P* = 0.020) in CARS (Fig. 1). This result implies that an abnormal level of plasma AVP is associated with visual experience and auditory sense impressions.

### Structural Differences Between ASD and TDC

**Fig. 2:**
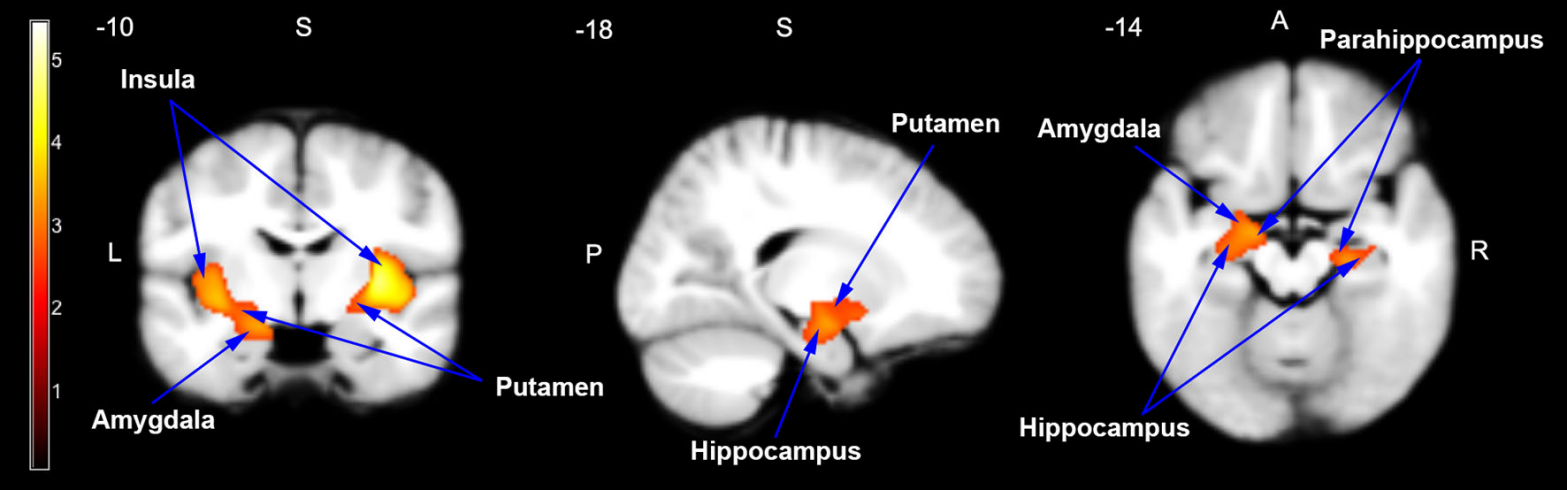
Enlarged brain areas in children with ASD compared to TDC in the coronal, sagittal, and axial planes. Two clusters were significantly larger in children with ASD than in TDC using voxel-based morphometric analysis. The clusters included the bilateral amygdala, hippocampus, parahippocampus, putamen, and insula. The enlarged brain areas are indicated by arrows on the customized pediatric template. *P* <0.01, corrected. The cluster in the left hemisphere was 2356 voxels, and the cluster in the right hemisphere was 3790 voxels.

In the VBM analyses, no significant differences were found in the raw volumes of GM, WM, CSF, and TIV between the ASD and TDC groups before and after covariates were controlled (Table 2). Voxel-wise whole-brain comparison showed that specific cortical and subcortical areas were significantly enlarged in the ASD group compared to the TDC group (*P* <0.01, corrected; cluster size >626 voxels): the bilateral insula, putamen, parahippocampus, hippocampus, and amygdala (Fig. 2).

**Table 2:** Tissue comparison of raw volume using voxel-based morphometric analysis.

Regions	Volume (cm^3^)				*F* score	*P* value	Corrected for covariates^e^	
	TDC		ASD					
	Mean	SD	Mean	SD			*F* score	*P* value
GM^a^	729.43	44.79	708.58	92.34	0.578	0.454	1.220	0.280
WM^b^	378.40	32.92	377.42	69.54	0.002	0.962	0.054	0.818
CSF^c^	165.47	28.69	158.47	31.80	0.374	0.546	0.173	0.681
TIV^d^	1273.30	88.34	1244.47	164.14	0.335	0.568	0.188	0.668

^a^GM, gray matter

^b^WM, white matter

^c^CSF, cerebrospinal fluid

^d^TIV, total intracranial volume

^e^covariates of additional analyses of age and gender; TDC, typically developing children; ASD, autism spectrum disorder.

Additional volume measurements of nuclei were then performed according to the voxel-based results, including the bilateral hypothalamus where AVP is synthesized as well as the bilateral amygdala and hippocampus as regions innervated by central AVP neurons. The results showed that the volumes of the left amygdala (1.39 ± 0.18 cm^3^ for ASD, 1.27 ± 0.11 cm^3^ for TDC; *F* = 4.520, *P* = 0.045) and the left hippocampus (3.54 ± 0.44 cm^3^ for ASD, 3.24 ± 0.39 cm^3^ for TDC; *F* = 4.548, *P* = 0.043) were significantly enlarged in children with ASD. When controlling for the effects of age and gender, the volume of the left amygdala remained significantly larger than that in TDC (*F* = 6.46, *P* = 0.018), but this was not seen in the left hippocampus (*F* = 4.019, *P* = 0.056). Moreover, the volumes of the bilateral hypothalamus were significantly lower (left: *F* = 13.126, *P* = 0.001; right: *F* = 15.253, *P* = 0.001) in the ASD group (left: 0.50 ± 0.07 cm^3^; right: 0.45 ± 0.05 cm^3^) than in the TDC group (left: 0.59 ± 0.06 cm^3^; right: 0.54 ± 0.07 cm^3^), even when the effects of age and gender were carefully controlled (left: *F* = 12.017, *P* = 0.002; right: *F* = 12.006, *P* = 0.002).

### Correlation Between Plasma AVP and Volume of Hypothalamus

**Fig. 3:**
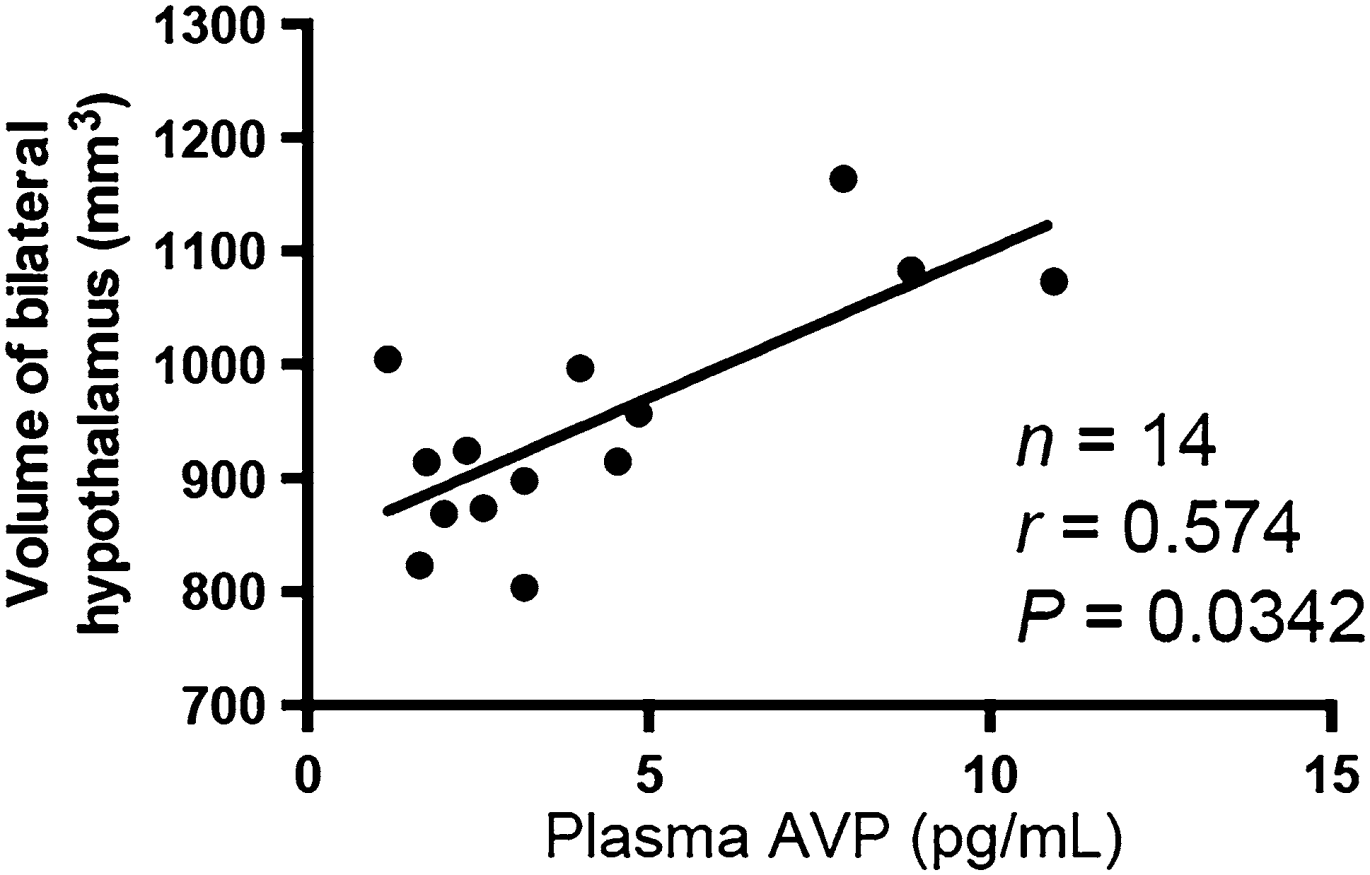
Correlation between plasma AVP level and the volume of the hypothalamus. The plasma level of AVP was positively correlated with the volume of the bilateral hypothalamus, indicating that a smaller hypothalamus may be predicted by a lower circulating level of AVP.

Spearman correlation analysis demonstrated that the plasma AVP concentration was positively correlated with the total volume of the hypothalamus (*r* = 0.574, *P* = 0.0342). This result suggests that the AVP level is closely related to the morphology of the hypothalamus (Fig. 3).

### Analyses of Functional Connectivity

#### Seed Point Set in the Left Amygdala

**Fig. 4:**
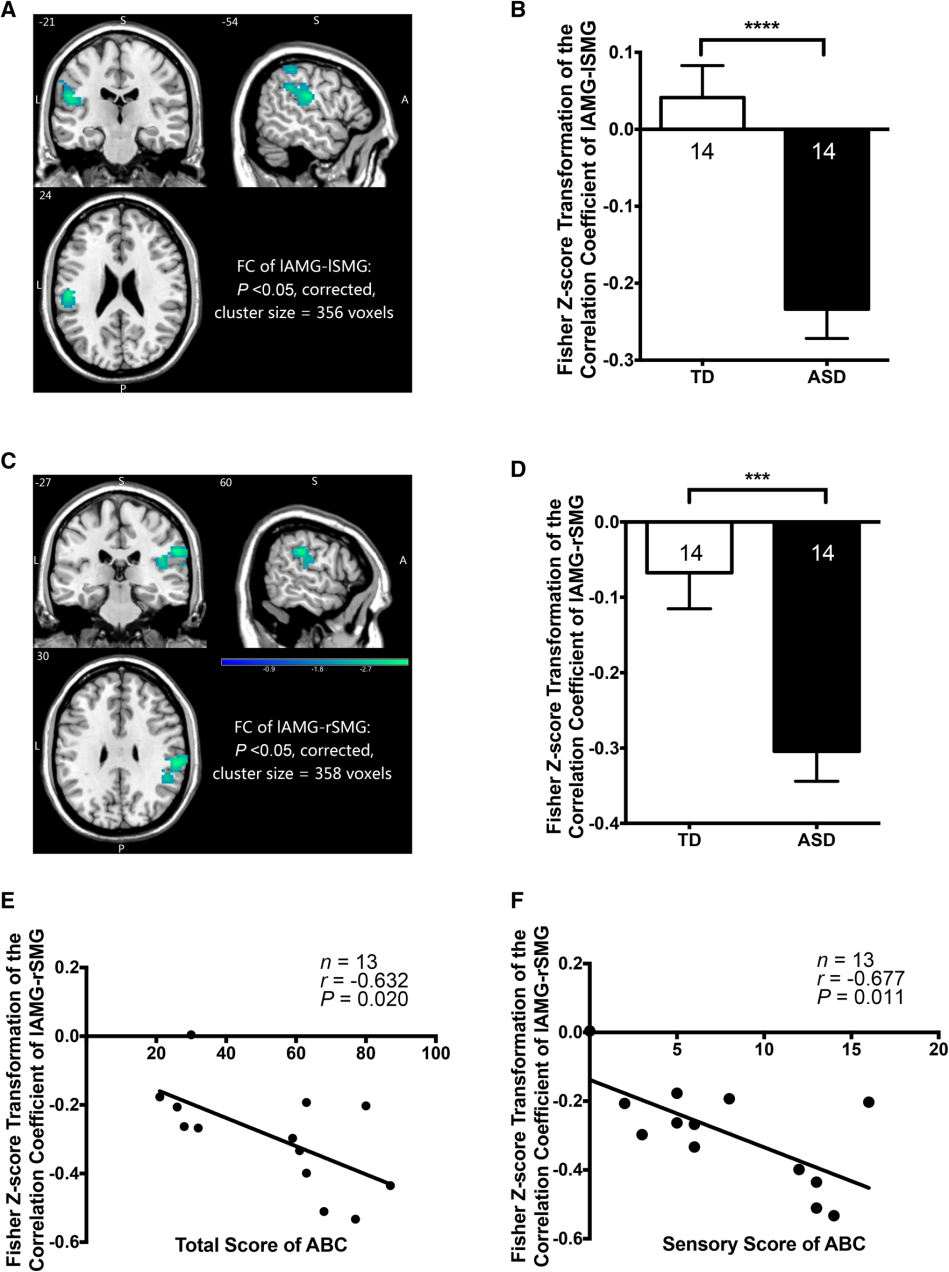
Functional connectivities (FCs) between left amygdala and bilateral supramarginal gyrus and correlations between the FCs and behavioral scores. **A**, **B** The FC of lAMG-lSMG showed a significantly stronger negative connection in children with ASD than the TDC (*P* <0.05, controlled for age and gender, corrected; cluster size, 390 voxels, peak MNI coordinates, −54, −18, 24). **C**, **D** The FC of lAMG-rSMG showed a significantly stronger negative connection in children with ASD than the TDC (*P* <0.05, controlled for age and gender, corrected; cluster size, 358 voxels; peak MNI coordinates, 60, −27, 30). **E**, **F** The FC of lAMG-rSMG was negatively correlated with both the total ABC score and the sensory ABC score. ABC, Autism Behavior Checklist; ASD, autism spectrum disorder; TDC, typically developing children; MNI, Montreal Neurological Institute; lAMG, left amygdala; lSMG, left supramarginal gyrus; rSMG, right supramarginal gyrus.

The voxel-wise analyses revealed that both the right supramarginal gyrus (rSMG; *P* <0.05, corrected; cluster size, 358 voxels; peak MNI coordinates, 60, −27, 30) and left supramarginal gyrus (lSMG; *P* <0.05, corrected; cluster size, 356 voxels; peak MNI coordinates, −54, −21, 24) showed significant negative FC to the left amygdala (lAMG) in children with ASD compared to TDC when controlled for age and gender (Fig. 4A–D). Correlation analyses revealed that in children with ASD, significant correlations existed between the FC of lAMG-rSMG and total ABC score (*r* = −0.632, *P =* 0.020), and between the FC of lAMG-rSMG and sensory ABC score (*r* = −0.677, *P =* 0.011) (Fig. 4E, F). A strong negative FC was associated with a high sensory score and the total ABC score.

#### Seed Point Set in the Left Hippocampus

**Fig. 5:**
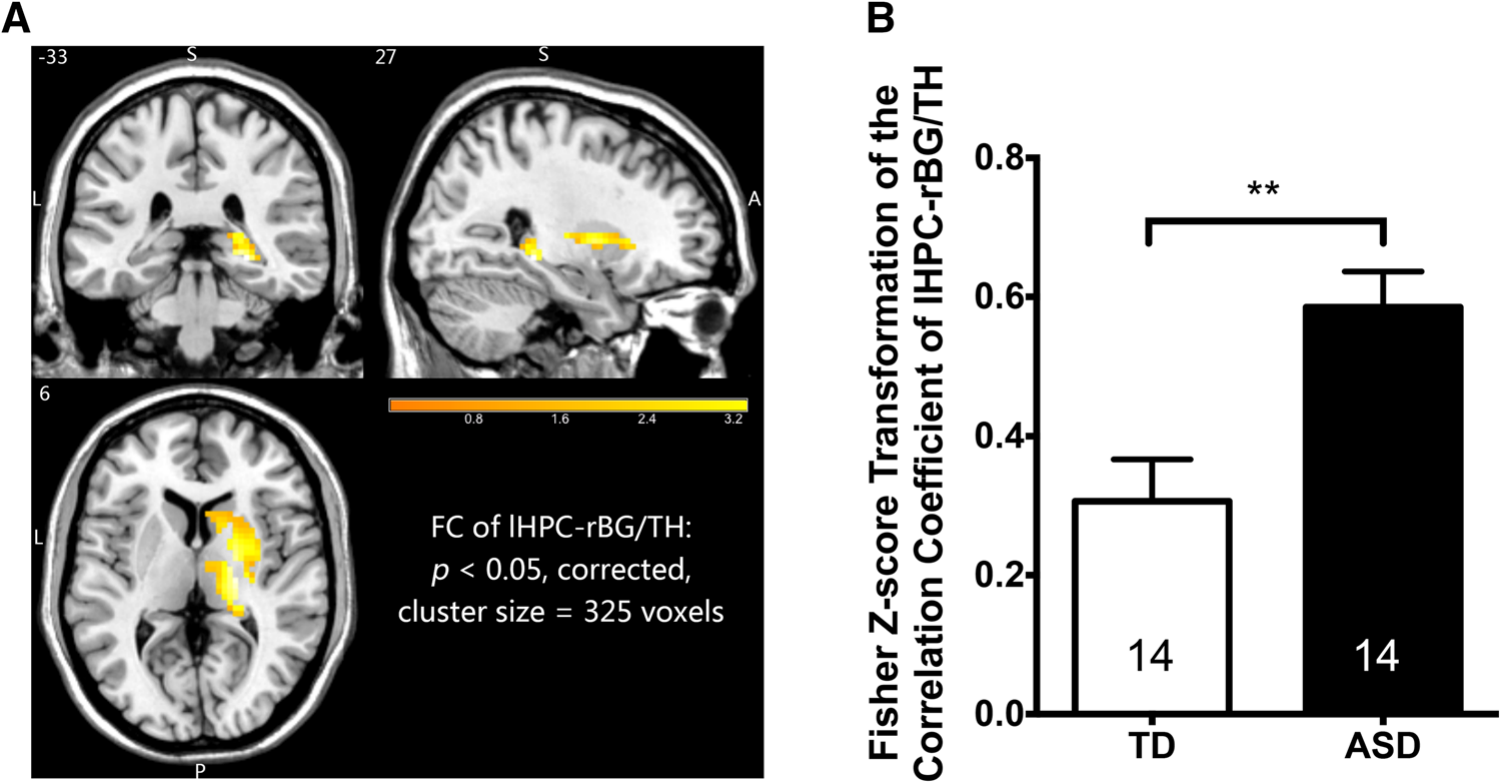
Functional connectivity between left hippocampus (lHPC) and right basal ganglia/thalamus (rBG/TH) was significantly higher in children with ASD than in TDC. *P* <0.05, controlled for age and gender, corrected; cluster size, 325 voxels; peak MNI coordinates, 27, −33, −6. ASD, autism spectrum disorder; TDC, typically developing children; MNI,Montreal Neurological Institute.

The right basal ganglia and right thalamic area (rBG/TH) were found to have a higher FC to the left hippocampus (lHPC) in children with ASD than TDC (*P <*0.05, corrected; cluster size, 325 voxels; peak MNI coordinates, 27, −33, −6) with age and gender set as covariates (Fig. 5).

### Correlations Between Brain Function and Plasma AVP

**Fig. 6:**
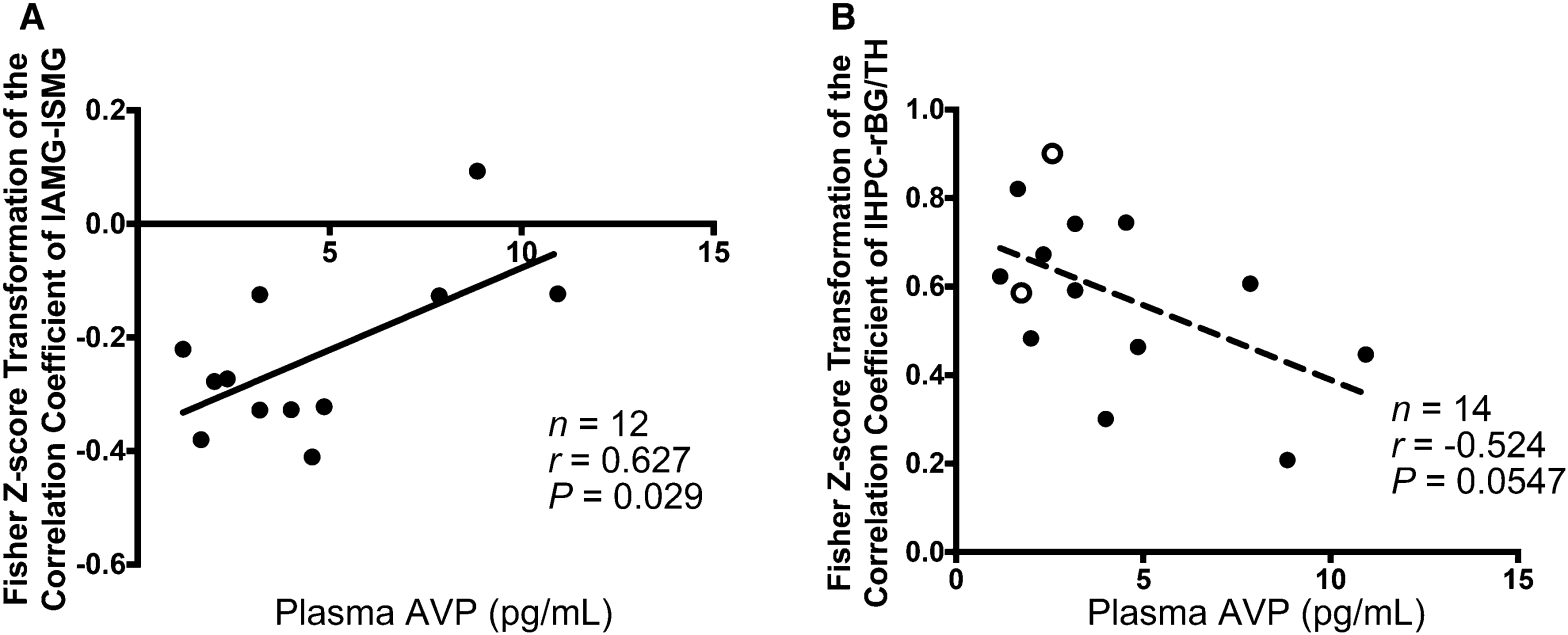
Correlations between brain functional connectivities (FCs) and plasma AVP concentrations in children with ASD. **A** FC of lAMG-lSMG was positively correlated with plasma AVP concentration in boys with ASD. **B** FC of lHPC-rBG/TH showed a strong trend for negative correlation with plasma AVP concentration in all children with ASD. lSMG, left supramarginal gyrus; lHPC, left hippocampus; rBG/TH, right basal ganglia/thalamus; AVP, arginine-vasopressin. *Filled circles* denote boys; *empty circles* denote girls.

We further examined the connection between brain function and plasma levels of AVP in autistic children. Pearson correlation analyses revealed that only in boys with ASD was there a significant positive correlation between the FC of lAMG-lSMG and plasma AVP concentration (*r* = 0.627, *P =* 0.029) (Fig. 6A). In all children with ASD, there was a tendency for a negative correlation between the FC of lHPC-rBG/TH and the plasma level of AVP (*r* = −0.524, *P =* 0.055) (Fig. 6B). These revealed that in children with ASD, especially boys, a lower plasma AVP level is associated with more aberrant FC.

### Structural Equation Model in a Hypothetical Pathway

**Fig. 7:**
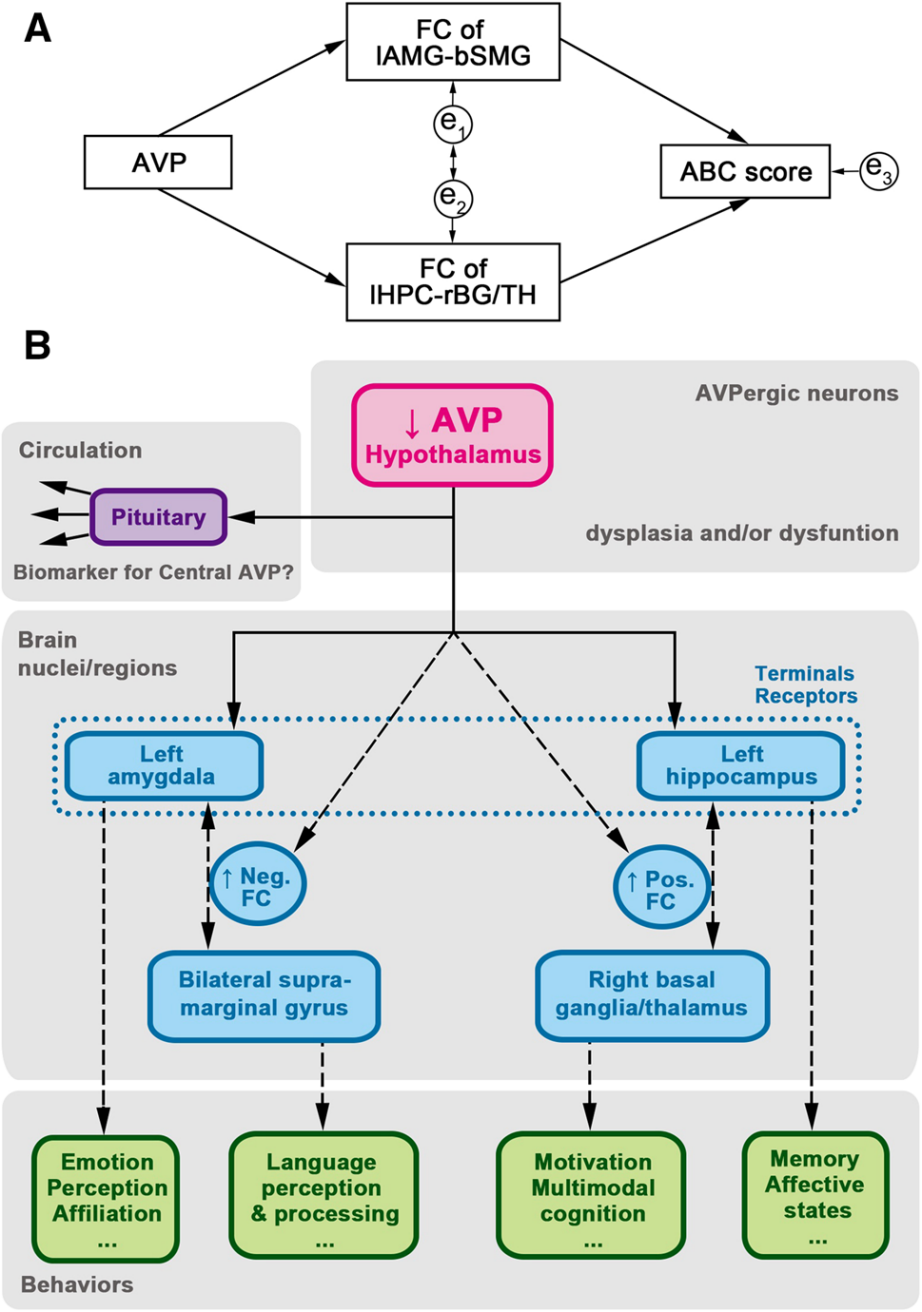
Diagram of hypothetical central AVP system pathway in children with ASD. (A) Hypothetical statistical model: χ^2^ = 0.233, *P* = 0.629, df = 1, RMSEA <0.001, GFI, 0.990; and AGFI, 0.905; e1-e3 indicate that errors could affect the model. (B) Diagram of hypothetical pathway. The blood AVP may serve as a biomarker of central AVP level. A descending AVP level impacts the function of the left amygdala and left hippocampus, which further extend their connectivity to other brain regions, resulting in various behavioral disorders. Solid lines with one-way arrows indicate secretion and projection. Broken lines with one-way arrows indicate a possible impact on function. Broken lines with two-way arrows indicate the possible functional connectivity between brain regions. AGFI, adjusted goodness-of-fit index; AVP, arginine-vasopressin; df, degrees of freedom; FC, functional connectivity; GFI, goodness-of-fit index; RMSEA, root mean square error of approximation.

Based on the above results, we constructed a hypothetical pathway in children with ASD (Fig. 7A). The FC value of lAMG-bSMG (bilateral supramarginal gyrus) was the average of the FC values of lAMG-rAMG and lAMG-lSMG. The results seem to fit with a satisfactory model of a pathway with the following data set: *χ* ^2^ = 0.233, *P* = 0.629, df = 1, RMSEA <0.001, GFI = 0.990, and AGFI = 0.905.

## Discussion

In the present study, we found evidence that the severity of ASD is associated with changes in the morphology and functionality of brain regions where AVP systems are involved. When compared to TDC, the children with ASD showed a decreased GM volume of the hypothalamus where the somata of AVP neurons are located, and an increased volume of the left amygdala and left hippocampus where the terminals of AVP-containing neurons reside. Additional functional analyses revealed a negative FC between the lAMG and bilateral SMG as well as an increased positive FC between the lHPC and rBG/TH in children with ASD. In addition, plasma level of AVP seemed to be useful for predicting the FC between the lAMG and lSMG in boys with ASD.

### Aberrant Morphology Suggests Dysfunction of the AVP/OXT Systems

Our data are in line with the results of several previous studies. For example, an enlarged amygdala and hippocampus in young children with ASD has been reported where endogenous AVP/OXT [6, 7] and administration of exogenous AVP/OXT seem to have a beneficial effect on children/patients with ASD [10, 46]. In a review, Courchesne and colleagues noted that the amygdala is one of the most markedly overgrown areas in children with autism [47]. Abnormal expansion of the amygdala is likely to occur before 2 years of age [48, 49] and the degree of abnormal expansion is positively correlated with the severity of social and communication impairment [49]. The hippocampus is also enlarged in children with autism, and the abnormal developmental pattern is considered to affect brain function [50–52].

The results of the present study show that volume of the hypothalamus, where the somata of the AVPergic and OXTergic neurons are located, is reduced in children with ASD, even when controlled for covariates. This finding was supported by the previous report that GM in the hypothalamus is diminished in children with autism [53]. Moreover, correlation analysis revealed that a smaller hypothalamic volume was associated with a lower plasma AVP concentration. The reduction in the volume of the hypothalamus might suggest dysplasia of neurons and/or neuropil in the hypothalamus that leads to a low level of AVP/OXT and the manifestation of autistic symptoms. Here, we found no significant difference in whole-brain volume between the ASD and TDC groups, suggesting that the volume changes in the hypothalamus, amygdala, and hippocampus were site-specific.

In addition, using imaging techniques, some genetic studies in humans have demonstrated that the most common phenotype of oxytocin receptor gene mutations (rs2254298 and rs53576) is shrinkage of the hypothalamus [54, 55] and enlargement of the amygdala [55–57]. These two gene mutations have been considered as candidate gene mutations for autism, especially in the Chinese population [58]. Thus, impairments in the AVP/OXT systems might be associated with aberrant brain morphology and function in autism.

### Negative Connectivity Between the Amygdala and Supramarginal Gyrus May Affect the Functionality of the “Social Brain”

The amygdala, a hub in the “social brain”, seems to play key roles in emotion, social cognition, perception, and affiliation [59]. The supramarginal gyrus, part of the mirror-neuron system in the “social brain”, is involved in language perception and processing [60]. It has been reported in a resting-state fMRI study that the amygdala had reduced functional connectivity within and between the default mode network incorporating “social brain” regions under ASD conditions in adults [61]. However, in the present study, the TDC showed extremely week connectivities between the left amygdala and bilateral supramarginal gyrus, which suggested there are different connective patterns in healthy adults and young children. Conversely, children with ASD showed an inhibitory connection between the left amygdala and bilateral supramarginal gyrus. This aberrant connective pattern may perceive information as aversive, causing failure of establishment of the social network. Furthermore, the FC seems to be negatively correlated with the autistic symptoms: a potentiation of the FC was associated with an augmentation of abnormal behavior and sensory deficit (Fig. 3E, F). It should be pointed out that the changes in connectivity and in GM volume occurred unilaterally in the present study. This is not an unusual phenomenon in connectivity and structural analysis using the fMRI technique since the left and right hemispheres are not identical in structure or function.

Animal experiments have shown that AVP has diverse effects on social behavior across a variety of mammalian species [62], suggesting that endogenous AVP may play a similar role in modulating social communications in humans. The present study revealed a strong association between circulating AVP level and lAMG-lSMG connections: a low plasma AVP concentration was associated with a strong negative lAMG-lSMG connection, a phenomenon that appeared only in boys with ASD. In the rodent, AVP immunoreactivity is more pronounced in the male brain, implying that AVP may contribute more in regulating male social behavior [63, 64]. These data indicate that the function of the amygdala, which is one of the major nuclei innervated by AVP-containing neurons from the hypothalamus, is sexually dimorphic, a well-recognized phenomenon seen in ASD.

### Hyper-Connectivity of the Hippocampus and Basal Ganglia/Thalamus May Imply Sustained Immature Brain Function

Apart from the well-known function of hippocampus in learning and memory, this region has also been implicated in the regulation of affective states and emotional behavior. It has also been implied to work through inhibitory connections with many subcortical structures to relieve the stress response [65]. The basal ganglia and thalamus, on the other hand, might play a critical role in the development of emotion, motivation, and multimodal cognition beyond simple sensorimotor function [66]. Similar to our study, some previous studies have found hyper-connectivity in subcortical regions [67–70] in ASD patients, such as the thalamus/basal ganglia and sensorimotor regions. Supekar and colleagues assumed that, in healthy individuals, the functional brain networks in childhood are more strongly connected between subcortical and paralimbic areas, whereas in adolescents and adults a stronger cortico-cortical connectivity may exist among the paralimbic, limbic and association areas [71]. Hence, the hyper-connectivity of the lHPC and rBG/TH may reflect developmental retardation of the autistic brain, which has been regarded as a specific feature in the ASD brain.

Moreover, AVP-containing neurons clearly play an active part in the connectivity between the lHPC and rBG/TH: a higher plasma AVP concentration might predict a lower connectivity between the IHPC and rBG/TH in children with ASD. However, the mechanism is still obscure and deserves further investigation.

### A Hypothesized Framework of the Central AVP System in Relation to Social Behavior

In summary, it is hypothesized that in children with ASD, abnormal development and dysfunction of the hypothalamus may impact the brain AVP system that leads to a state of stronger negative-connectivity between the lAMG and bilateral SMG (limbic-association area connectivity) and a state of hyper-connectivity between the lHPC and rBG/TH (limbic-subcortical area connectivity). These aberrant brain connections may eventually result in abnormal emotion, language and social interactions in children with ASD (Fig. 7B).

On the other hand, the peripheral AVP level may be taken as a predictive index to reflect the central AVP level and related symptoms in children with ASD. Carson and colleagues found that AVP concentrations in plasma and CSF are positively correlated in children [31, 32]; lower plasma AVP concentration is correlated with lower social function [32]. Nevertheless, the relationship between central and peripheral AVP levels remains controversial. The results may be influenced by the age of participants and their physiological status during sample collection. In the present study, the age of the participant children had a relatively narrow range and a strictly-controlled dietary status during blood collection. The results showed that the plasma AVP concentration was significantly correlated with the behaviors, hypothalamic volume, and functional connectivities. Besides, our previous interventional study demonstrated that an increased plasma AVP level was positively correlated with behavioral improvement in children with ASD [30]. These data suggest that the peripheral level of AVP may reflect that of the central level.

### Limitations and Future Directions

In the present study, efforts were made to explore the differences in brain function between children with ASD and TDC following sedation with oral chloral hydrate. Chloral hydrate is a safe and commonly-used sedative during pediatric examinations, such as MRI and CT, since sometimes it is difficult to complete the procedures under awake or natural sleep conditions, especially in toddlers and preschoolers. There is always a concern whether the abnormalities are artifacts of chloral hydrate. There are only limited studies in literature to show the effects of chloral hydrate on brain activity. Rodent experiments have shown that normal low-frequency fluctuations in the BOLD signal can be detected [72] and visual and auditory cortices can be activated in children with chloral hydrate-induced sedation [73], suggesting that brain function can be studied under chloral hydrate sedation in certain individuals. Though the inter-hemispheric FC has been shown to be lower in mice treated with alpha-chloralose than in awake mice [74], we believe that the major differences in connectivity detected in the present study were not attributable to chloral hydrate since the TDC group was sedated in the same way. However, caution should be taken when interpreting the data since natural sleep and chloral hydrate-assisted sleep are not the same.

Our sample size was relatively small and future studies with larger sample sizes are warranted to confirm the results with greater confidence. The blood collection and analysis of plasma AVP were done only in the autistic children who had completed the MRI scanning. The interpretation of the data would be more convincing if plasma AVP levels had also been obtained from TDC.

In addition, we focused only on abnormalities of the AVP system in ASD. It would be interesting to investigate both AVP and OXT in future studies, and explore the possible interactions between these two neuropeptides and brain function.

## Conclusions

To the best of our knowledge, this is the first study to explore the links among circulating AVP, brain imaging, and behaviors in young children with ASD. The hypothalamus on both sides was found to be shrunken, and this was associated with a lower AVP level. Besides, the left amygdala and left hippocampus were enlarged in children with ASD. All these findings indicate an aberrant morphology of AVPergic neurons. Stronger negative connectivity between the left amygdala and bilateral supramarginal gyrus and stronger positive connectivity between the left hippocampus and right basal ganglia/thalamus were found in children with ASD compared to TDC. These aberrant FCs revealed in autistic children seem to be associated with the degree of severity of symptoms and reduction of plasma AVP. Moreover, AVP might be one of the neurochemical candidates that affect certain brain functions and in turn affect autistic behaviors. These findings may be beneficial for understanding the etiology and neurobiological basis of ASD.

## Electronic supplementary material

Below is the link to the electronic supplementary material.

Supplementary material 1 (PDF 746 kb)
