# Supplementary material for: A Volumetric and Functional Connectivity MRI Study of Brain Arginine-Vasopressin Pathways in Autistic Children
Source: Neurosci Bull. 2017 Mar 3;33(2):130–42. doi: 10.1007/s12264-017-0109-2 (PMC5360858; doi:10.1007/s12264-017-0109-2)
Supplement: Supplementary file 1 — Supplementary material 1 (PDF 746 kb) [file 12264_2017_109_MOESM1_ESM.pdf]

Supplemental material

A Volumetric and Functional Connectivity MRI Study of Brain Arginine-vasopressin Pathways in Autistic Children

Xiao-Jing Shou, Xin-Jie Xu, Xiang-Zhu Zeng, Ying Liu, Hui-Shu Yuan, Yan Xing, Mei-Xiang Jia, Qing-Yun Wei, Song-Ping Han, Rong Zhang,

Ji-Sheng Han

Table S1. Demographics and scale characteristics in children with ASD

Subjects	Age (years)	Gender	CARS score	ABC score	AQ-Child score
ASD01	5.0	Female	30.0	87	37
ASD02	3.7	Male	41.0	80	23
ASD03	4.8	Male	30.0	63	22
ASD04	5.0	Male	30.5	21	19
ASD05[a]	4.0	Male	-	-	-
ASD06	4.0	Male	39.5	63	27
ASD07	3.4	Male	41.0	61	35
ASD08	2.9	Female	37.0	26	42
ASD09	4.5	Male	38.0	28	29
ASD10	3.1	Male	33.0	32	32
ASD11	3.5	Male	37.5	77	34
ASD12	5.0	Male	34.0	68	31
ASD13	4.5	Male	32.5	30	24
ASD14	4.0	Male	36.0	59	33

[a]This child missed the CARS assessment and his parent questionnaire was technically invalid.

CARS, Childhood Autism Rating Scale; ABC, Autism Behavior Checklist; AQ-Child, Autism Spectrum Quotient Children's Version; ASD, autism spectrum disorder.

The normality of the characteristics (except for gender) was tested by the Shapiro-Wilk test.

Table S2. Demographics and scale characteristics in TDC

Subjects	Age (years)	Gender	ABC score	AQ-Child score
TDC01	3.5	Female	0	9
TDC02	5.5	Male	2	18
TDC03	5.0	Male	0	13
TDC04	5.0	Male	5	16
TDC05	5.0	Male	37	18
TDC06	4.0	Male	11	13
TDC07	4.0	Female	4	20
TDC08	4.0	Male	15	19
TDC09	5.0	Female	3	13
TDC10	4.0	Male	3	24
TDC11	3.0	Male	12	20
TDC12	5.0	Male	20	17
TDC13	5.2	Female	23	14
TDC14	4.8	Male	15	28

CARS, Childhood Autism Rating Scale; ABC, Autism Behavior Checklist; AQ-Child, Autism Spectrum Quotient Children's Version; TDC, typically developing children.

The normality of the characteristics (except for gender) were tested by the Shapiro-Wilk test.

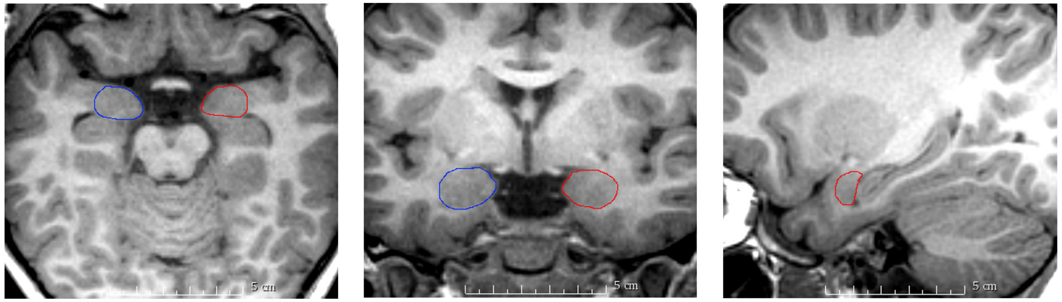

Fig. S1. Amygdala manual tracing in axial, coronal and sagittal planes (red, left; blue, right) in one participant's original-space brain. The amygdala is located within the temporal lobes, the superior aspect is shown to be partly continuous with the inferior margin of the claustrum, separated from the inferior putamen, and in close contact with the optic tract [1, 2].

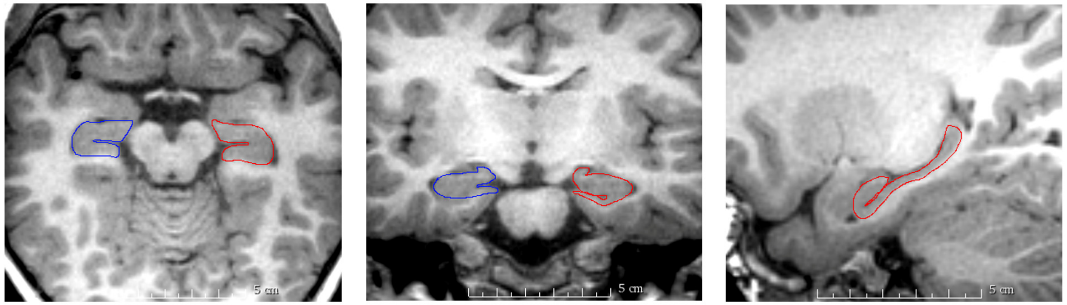

Fig. S2. Hippocampus manual tracing in axial, coronal and sagittal planes (red, left; blue, right) in one participant's original-space brain. The hippocampus is located in the mesial temporal lobe and protrudes into the temporal horn of the lateral ventricle. Its rostral extremity extends ventrally to the amygdala [1, 3]

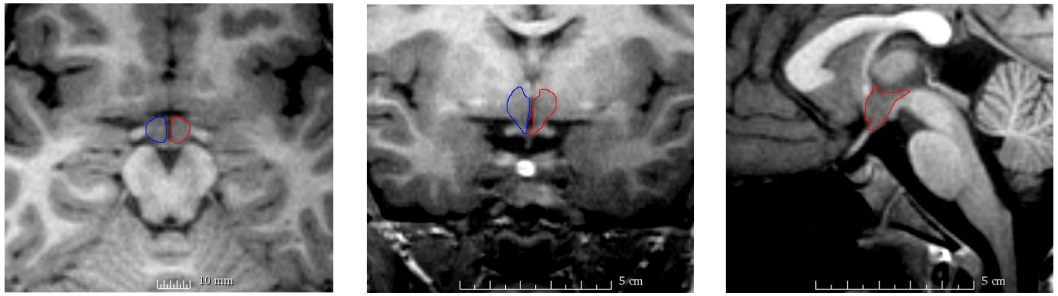

Fig. S3. Hypothalamus manual tracing in axial, coronal and sagittal planes (red, left; blue, right) in one participant's original-space brain. The hypothalamus lies ventral to the thalamic sulcus, extending from the lamina terminalis at the chiasmal notch in the region of the optic chiasma through a vertical plane caudal to the mammillary body [1, 4]

REFERENCES

1. Tamraz, J.C., et al., *Atlas of regional anatomy of the brain using MRI with functional correlations*. 2006, Springer: Berlin. p. 1 v.
2. Ortiz-Mantilla, S., et al., *Associations between the size of the amygdala in infancy and language abilities during the preschool years in normally developing children.* Neuroimage, 2010. **49**(3): p. 2791-9.
3. Schumann, C.M., et al., *The amygdala is enlarged in children but not adolescents with autism; the hippocampus is enlarged at all ages.* J Neurosci, 2004. **24**(28): p. 6392-401.
4. Lemaire, J.J., et al., *Maps of the adult human hypothalamus.* Surg Neurol Int, 2013. **4**(Suppl 3): p. S156-63.
